# Terminal Epitope-Dependent Branch Preference of Siglecs Toward *N*-Glycans

**DOI:** 10.3389/fmolb.2021.645999

**Published:** 2021-04-29

**Authors:** Shuaishuai Wang, Congcong Chen, Minhui Guan, Ding Liu, Xiu-Feng Wan, Lei Li

**Affiliations:** ^1^ Department of Chemistry, Georgia State University, Atlanta, GA, United States; ^2^ MU Center for Influenza and Emerging Infectious Diseases, University of Missouri, Columbia, MO, United States; ^3^ Department of Molecular Microbiology and Immunology, School of Medicine, University of Missouri, Columbia, MO, United States; ^4^ Bond Life Sciences Center, University of Missouri, Columbia, MO, United States; ^5^ Department of Electrical Engineering and Computer Science, College of Engineering, University of Missouri, Columbia, MO, United States

**Keywords:** Siglecs, asymmetric *N*-glycan, Neu5Gc, Neu5Ac, microarray

## Abstract

Siglecs are sialic acid–binding immunoglobulin-like lectins that play vital roles in immune cell signaling. Siglecs help the immune system distinguish between self and nonself through the recognition of glycan ligands. While the primary binding specificities of Siglecs are known to be divergent, their specificities for complex glycans remain unclear. Herein, we determined *N*-glycan binding profiles of a set of Siglecs by using a complex asymmetric *N*-glycan microarray. Our results showed that Siglecs had unique terminal epitope-dependent branch preference when recognizing asymmetric *N*-glycans. Specifically, human Siglec-3, -9, and -10 prefer the α1-3 branch when Siaα2-6Galβ1-4GlcNAc terminal epitope serves as the binding ligand but prefer the opposite α1-6 branch when Siaα2-3Galβ1-4GlcNAc epitope serves as the ligand. Interestingly, Siglec-10 exhibited dramatic binding divergence toward a pair of Neu5Ac-containing asymmetric *N*-glycan isomers, as well as their Neu5Gc-containing counterparts. This new information on complex glycan recognition by Siglecs provides insights into their biological roles and applications.

## Introduction

Sialic acid–binding immunoglobulin-like lectins (Siglecs) are cell-surface transmembrane receptors that are differentially expressed on immune cells (Läubli and Varki, 2020). They play critical roles in immune cell signaling and help the immune system to distinguish self and nonself (Macauley et al., 2014). Most Siglecs, with the only exception being sialoadhesin/Siglec-1, have C-terminal regulatory motifs in their cytoplasmic domains that participate in the regulation of immune systems. On the *N-*terminal, each Siglec has a V-set immunoglobulin (Ig) domain that recognizes sialic acid–containing glycans (Duan and Paulson, 2020). There are 15 human Siglecs and 9 murine Siglecs. Among those, four are conserved across mammals (Siglec-1, 2, 4, and 15). All remaining Siglecs are named CD33-related Siglecs as they contain less conserved structure between humans and other vertebrates, but all have high homologies to CD33.

Siglecs are immune-modulatory receptors within the mammalian immune system. Most Siglecs have intracellular immunoreceptor tyrosine inhibitory motifs (ITIMs) that can, in principle, participate in inhibitory or activating signals. The binding of anti-Siglec antibodies or multivalent trans-ligand with inhibitory Siglecs can activate/phosphorylate the ITIMs and produce negative signals (Duan and Paulson, 2020). Additionally, some Siglecs are specifically expressed on certain types of immune cells and presented as endocytic receptors. Hence, they were utilized as the desired target for drug development. For example, Siglec-3, also called CD33, is an inhibitory receptor that is relatively specifically expressed on myeloid lineage and endocytosed upon antibody binding, thus serving as a specific target for developing therapeutic antibodies. Gemtuzumab ozogamicin is the first approved CD33-targeting antibody-drug conjugate (ADC) and was used for induction therapy of acute myeloid leukemia (AML) (Laszlo et al., 2014).

Despite the diverse roles that Siglecs play in immune cell regulation and disease processes, their natural ligands, especially the fine binding specificity, toward complex glycans are relatively underinvestigated. Glycan microarray was developed for identifying interactions between glycans and glycan-binding proteins (GBPs) 2 decades ago (Fukui et al., 2002; Palma and Chai, 2019). It enabled simultaneous binding analysis of GBPs to hundreds of glycan structures and had become a major tool to unveil glycan–protein interactions (Gao et al., 2019b). Various versions of glycan microarray were used to investigate interactions between glycans and Siglecs (Blixt et al., 2003; Bochner et al., 2005; Campanero-Rhodes et al., 2006; Rillahan et al., 2012; Rillahan et al., 2013; Gao et al., 2019a). However, the fine specificity details of Siglecs toward natural complex glycans remain largely unknown.

Herein, we investigated the binding specificity of Siglec-3, -9, -10, and -F using a unique glycan microarray containing 98 structurally well-defined complex glycans, revealing a unique terminal epitope-dependent branch preference toward asymmetric *N*-glycans. Particularly, a dramatic binding divergence of Siglec-10 toward a pair of *N*-glycan isomers was observed and further confirmed by synthesized Neu5Gc-containing counterparts. Later, quantitative assay by biolayer interferometry analyses suggested a 67-fold avidity difference among the Neu5Gc-containing isomers.

## Materials and Methods

Unless otherwise stated, all chemicals were purchased and used without further purification. The 98 *N*-glycan microarray was prepared as described previously (Supplementary Figure S1) (Li et al., 2019). Sugar nucleotides, including uridine 5′-diphospho-galactose (UDP-Gal) (Muthana et al., 2012), were prepared as described previously. Enzymes including *Neisseria meningitides* β1-4galactosyltransferase (NmLgtB) (Lau et al., 2010), *N. meningitidis* CMP-sialic acid synthetase (NmCSS) (Yu et al., 2004), *Pasteurella multocida* α2-3sialyltransferase mutant M144D (PmST1-M144D) (Sugiarto et al., 2012), and *Photobacterium damsela* α2-6sialyltransferase (Pd26ST) (Yu et al., 2006) were expressed and purified as previously described.

### Chemoenzymatic Synthesis of *N*-Glycans

*N*-glycans **38** and **54** were prepared as previously reported (Li et al., 2015). For the α2-6sialylation of **38**, **100**, **54**, and **104**, reactions were carried out in reaction systems containing Tris-HCl (100 mM, pH 8.0), an acceptor glycan (10 mM), CTP (15 mM), *N*-acetylneuraminic acid (Neu5Ac) or *N*-glycolylneuraminic acid (Neu5Gc) (15 mM), MgCl_2_ (10 mM), and appropriate amounts of NmCSS and Pd26ST. Reactions were incubated at 37°C for 3 h and monitored by HPLC. After over 95% acceptor was converted, reactions were quenched by the addition of equal volumes of ice-cold ethanol, concentrated, and subject to HPLC separation to afford compounds **99**, **101**, **103**, and **105**. Product-containing fractions were pooled and lyophilized for characterization and next step modular assembly. For the β1-4galactosylation of **99** and **103**, reactions were performed in mixtures containing Tris-HCl (100 mM, pH 7.5), an acceptor glycan (10 mM), UDP-Gal (15 mM), MgCl_2_ (10 mM), and an appropriate amount of NmLgtB. Reactions were incubated at 37°C overnight and monitored by HPLC. After over 95% acceptor was converted, reactions were quenched, concentrated, and subject to HPLC separation of compounds **100** and **104**. Product-containing fractions were pooled and lyophilized for characterization and subsequent synthesis. The α2-3sialylation of **100** and **104** was carried out in reaction systems containing Tris-HCl (100 mM, pH 8.0), an acceptor glycan (10 mM), CTP (15 mM), Neu5Gc (15 mM), MgCl_2_ (10 mM), and appropriate amounts of NmCSS and PmST1-M144D. PmST1-M144D-catalyzed reactions were incubated at 37°C for 3 h and monitored by HPLC. After over 90% acceptor was converted, the reaction was quenched, concentrated, and subject to HPLC separation to afford compounds **102** and **106**. Product-containing fractions were then pooled and lyophilized for characterization.

Newly synthesized *N*-glycans were purified by HPLC using a Waters XBridge BEH amide column (130 Å, 5 μm, 10 mm × 250 mm) under a gradient running condition (solvent A: water or 100 mM ammonium formate; solvent B: acetonitrile; flow rate: 4.5 ml/min, B%: 65–50% in 30 min) and monitored by UV absorbance at 210 nm. MALDI-TOF MS analyses were performed on UltrafleXtreme MALDI TOF/TOF Mass Spectrometer (Bruker). Scan range of MS was set according to molecular weight, and reflector mode was used for analysis. Mass spectra were obtained in negative extraction mode with the following voltage settings: ion source 1 (19.0 kV), ion source 2 (15.9 kV), and lens (9.3 kV). The reflector voltage was set to 20 kV. The laser was pulsed at 7 Hz and the pulsed ion extraction time was set at 400 ns. The laser power was kept in the range of 40–90%. ^1^H NMR spectra were recorded on a Bruker AVANCE 600 (600 MHz) spectrometer at 25°C. All ^1^H Chemical shifts (in ppm) were assigned according to D_2_O (δ = 4.79 ppm).

Compound **99**, white power (0.92 mg). ^1^H NMR (600 MHz, D_2_O) δ 5.05 (s, 1H), 4.83 (d, *J* = 1.7 Hz, 1H), 4.52 (dd, *J* = 8.0, 2.8 Hz, 2H), 4.47 (d, *J* = 8.4 Hz, 1H), 4.36 (d, *J* = 7.9 Hz, 1H), 4.17 (d, *J* = 1.9 Hz, 1H), 4.11 (dd, *J* = 3.5, 1.6 Hz, 1H), 4.02 (d, *J* = 7.3 Hz, 2H), 3.95–3.29 (m, 42H), 2.60 (dd, *J* = 12.4, 4.7 Hz, 1H), 2.02–1.92 (m, 9H), 1.65 (t, *J* = 12.2 Hz, 1H). MALDI-MS: C_67_H_111_N_5_O_50_, calc. for 1785.6297, found [M-H]^−^ 1784.765.

Compound **100**, white power (0.74 mg). ^1^H NMR (600 MHz, D_2_O) δ 5.10 (d, *J* = 2.7 Hz, 1H), 5.05 (s, 1H), 4.85 (d, *J* = 1.7 Hz, 1H), 4.54–4.46 (m, 3H), 4.38 (dd, *J* = 13.8, 7.9 Hz, 2H), 4.17 (s, 1H), 4.11 (dd, *J* = 3.6, 1.6 Hz, 1H), 4.03 (d, *J* = 4.4 Hz, 3H), 3.97–3.35 (m, 56H), 2.60 (dd, *J* = 12.4, 4.7 Hz, 1H), 2.03–1.91 (m, 11H), 1.65 (t, *J* = 12.2 Hz, 1H). MALDI-MS: C_73_H_121_N_5_O_55_, calc. for 1947.6825, found [M-H]^−^ 1946.883.

Compound **101**, white power (0.61 mg). ^1^H NMR (600 MHz, D_2_O) δ 5.10 (d, *J* = 2.8 Hz, 1H), 5.05 (s, 1H), 4.86 (s, 1H), 4.55–4.48 (m, 3H), 4.36 (d, *J* = 7.9 Hz, 2H), 4.17 (s, 1H), 4.11 (dd, *J* = 3.6, 1.5 Hz, 1H), 4.03 (s, 3H), 3.95–3.35 (m, 65H), 2.59 (ddd, *J* = 12.2, 7.4, 4.7 Hz, 2H), 2.03–1.91 (m, 15H), 1.64 (q, *J* = 11.8 Hz, 2H). MALDI-MS: C_84_H_138_N_6_O_63_, calc. for 2238.7779, found [M-2H + Na]^−^ 2259.918.

Compound **102**, white power (0.36 mg). ^1^H NMR (600 MHz, D_2_O) δ 5.05 (s, 1H), 4.84 (s, 1H), 4.55–4.43 (m, 4H), 4.36 (d, *J* = 7.9 Hz, 1H), 4.17 (d, *J* = 2.8 Hz, 1H), 4.11 (d, *J* = 3.4 Hz, 1H), 4.03 (s, 6H), 3.95–3.36 (m, 72H), 2.69 (dd, *J* = 12.4, 4.6 Hz, 1H), 2.60 (dd, *J* = 12.4, 4.7 Hz, 1H), 2.01–1.91 (m, 12H), 1.73 (t, *J* = 12.2 Hz, 1H), 1.65 (t, *J* = 12.2 Hz, 1H). MALDI-MS: C_84_H_138_N_6_O_64_, calc. for 2254.7728, found [M-2H + Na]^−^ 2276.097.

Compound **103**, white power (1.21 mg). ^1^H NMR (600 MHz, D_2_O) δ 5.10 (d, *J* = 2.6 Hz, 1H), 5.03 (s, 1H), 4.86 (s, 1H), 4.52 (t, *J* = 6.4 Hz, 2H), 4.47 (d, *J* = 8.4 Hz, 1H), 4.37 (d, *J* = 7.9 Hz, 1H), 4.17 (s, 1H), 4.10 (dd, *J* = 3.4, 1.6 Hz, 1H), 4.03 (s, 3H), 3.95–3.32 (m, 56H), 2.60 (dd, *J* = 12.3, 4.7 Hz, 1H), 2.02–1.91 (m, 12H), 1.65 (t, *J* = 12.2 Hz, 1H). MALDI-MS: C_67_H_111_N_5_O_50_, calc. for 1785.6297, found [M-H]^−^ 1784.867.

Compound **104**, white power (0.87 mg). ^1^H NMR (600 MHz, D_2_O) δ 5.10 (d, *J* = 2.7 Hz, 1H), 5.03 (s, 1H), 4.86 (d, *J* = 1.8 Hz, 1H), 4.55–4.48 (m, 3H), 4.37 (t, *J* = 7.8 Hz, 2H), 4.16 (d, *J* = 2.5 Hz, 1H), 4.11 (dd, *J* = 3.4, 1.6 Hz, 1H), 4.03 (d, *J* = 3.6 Hz, 3H), 3.95–3.37 (m, 55H), 2.60 (dd, *J* = 12.4, 4.7 Hz, 1H), 2.03–1.93 (m, 12H), 1.65 (t, *J* = 12.2 Hz, 1H), 1.24 (d, *J* = 6.9 Hz, 1H). MALDI-MS: C_73_H_121_N_5_O_55_, calc. for 1947.6825, found [M-H]^−^ 1947.017.

Compound **105**, white power (0.59 mg). ^1^H NMR (600 MHz, D_2_O) δ 5.10 (d, *J* = 2.8 Hz, 1H), 5.05 (s, 1H), 4.86 (s, 1H), 4.56–4.48 (m, 3H), 4.39–4.32 (m, 2H), 4.17 (s, 1H), 4.11 (dd, *J* = 3.5, 1.6 Hz, 1H), 4.03 (s, 3H), 3.95–3.38 (m, 58H), 2.59 (td, *J* = 12.9, 4.6 Hz, 2H), 2.04–1.90 (m, 12H), 1.64 (td, *J* = 12.2, 9.2 Hz, 2H). MALDI-MS: C_84_H_138_N_6_O_63_, calc. for 2238.7779, found [M-2H + Na]^−^ 2260.103.

Compound **106**, white power (0.43 mg). ^1^H NMR (600 MHz, D_2_O) δ 5.10 (d, *J* = 2.7 Hz, 1H), 5.03 (s, 1H), 4.86 (s, 1H), 4.54–4.44 (m, 4H), 4.37 (d, *J* = 7.9 Hz, 1H), 4.17 (d, *J* = 2.5 Hz, 1H), 4.14–4.09 (m, 1H), 4.03 (s, 6H), 3.94–3.38 (m, 40H), 2.69 (dd, *J* = 12.4, 4.6 Hz, 1H), 2.60 (dd, *J* = 12.4, 4.7 Hz, 1H), 2.01–1.94 (m, 12H), 1.73 (t, *J* = 12.2 Hz, 1H), 1.65 (t, *J* = 12.2 Hz, 1H). MALDI-MS: C_84_H_138_N_6_O_64_, calc. for 2254.7728, found [M-2H + Na]^−^ 2276.203.

### Glycan Derivatization and Quantification

All synthesized glycans with free reducing-end were derivatized by reductive amination using 2-amino-*N*-(2-aminoethyl)-benzamide (AEAB) as previously described (Song et al., 2009). Labeled glycans were further purified by HPLC to homogeneity using a porous graphitic carbon column (5 μm, 4.6 mm × 150 mm) under a gradient running condition (solvent A: 0.1% TFA in water; solvent B: 0.1% TFA in acetonitrile; flow rate: 1 ml/min, B%: 15–45% in 30 min) and monitored by UV absorbance at 330 nm. Product-containing fractions were pooled and lyophilized. The quantifications of AEAB-labeled glycans were conducted as previously described (Li et al., 2019).

### Neu5Gc-*N*-glycan Microarray Fabrication

The AEAB labeled–glycans were prepared at a concentration of 100 μM in the printing buffer (150 mM phosphate, pH 8.5), and printed on multivalent NHS-derivatized microscope-glass slides (Z Biotech, LLC), each for 400 pL in replicates of six, as described previously (Heimburg-Molinaro et al., 2011). Noncontact printing was performed at room temperature with a humidity of 60% by a sciFLEXARRAYER S3 spotter (Scienion) with two PDC 80 Piezo Dispense Capillary. After overnight dehumidification under room temperature, the slides were washed with MilliQ water and subsequently blocked with 50 mM ethanolamine in 100 mM Tris-HCl (pH 9.0) for 2 h. The blocked slides were then washed with MilliQ water twice, dried, and stored desiccated at −20°C until use.

### Microarray Assay of Siglecs With *N*-Glycan Microarrays

The 98 *N*-glycan microarray slide (Li et al., 2019) was fitted with a ProPlate 8-well microarray module (Sigma-Aldrich), and the subarrays were then rehydrated for 10 min with 200 μL of Buffer TSMTB (20 mM Tris-HCl, pH 7.4, 150 mM NaCl, 2 mM CaCl_2_, 2 mM MgCl_2_, 0.05% (v/v) Tween-20, and 1% (w/v) BSA) at room temperature. Then, the buffer was drained and 200 μL of Siglec-3, -8, -9, -10, and -F (R&D Systems) (20 µg/ml) in TSMTB were added into each subarray, sealed, and incubated at room temperature for 1 h with gentle shaking. Slides were then washed with Buffer TSMT (20 mM Tris-HCl, pH 7.4, 150 mM NaCl, 2 mM CaCl_2_, 2 mM MgCl_2_, and 0.05% (v/v) Tween-20) for four times. Next, 200 μL of 5 µg/ml goat anti-human IgG Fc antibody cross-adsorbed, DyLight^®^ 650 (Thermo Fisher) was added into each subarray, sealed, and incubated at room temperature for 1 h with gentle shaking. Finally, slides were washed with TSMT, TSM (20 mM Tris-HCl, pH 7.4, 150 mM NaCl, 2 mM CaCl_2_, and 2 mM MgCl_2_) and MilliQ water, four times for each buffer, respectively, and dried by brief centrifugation. Slides were scanned at a resolution of 10 μm using a Genepix 4100 A microarray scanner (Molecular Devices Corp) with 500 or 600 PMT gains and 80% power. Image analyses were carried out using Genepix Pro 6.0 as previously reported (Li et al., 2019). Spots were defined as circular features with a variable radius as determined by the Genepix scanning software, and local background subtraction was performed. Similarly, Siglec-10 was analyzed using the newly fabricated Neu5Gc-*N*-glycan array at concentrations of 1 and 5 μg/ml.

### Biolayer Interferometry Receptor Binding Assay and Data Analysis

The AEAB-labeled glycan **102** and **106** were labeled with Biotin by using the reagent EZ-Link™ NHS-Biotin (Thermo Fisher). In detail, 1 mM AEAB-labeled glycan was incubated with 10 mM NHS-Biotin at room temperature for 10 min. Then, labeled glycans were purified by HPLC to homogeneity using an ODS4 column (5 μm, 4.6 mm × 150 mm) under a gradient running condition (solvent A: 0.1% TFA in water; solvent B: 0.1% TFA in acetonitrile; flow rate: 1 ml/min, B%: 5–50% in 30 min), monitored by UV absorbance at 330 nm. Product-containing fractions were pooled and lyophilized for storage. The purified Biotin-labeled glycans were quantified by HPLC as described above.

Avidities were measured by biolayer interferometry using an Octet RED instrument (Pall FortéBio, Fremont, CA, United States). The prepared biotinylated glycans were preloaded onto streptavidin-coated biosensors at up to 100 nM for 3 min in 1× kinetic buffer (Pall FortéBio, Menlo Park, CA, United States). Siglec-10 was diluted to concentrations of 1 μM, 500 nM, and 250 nM with 1× kinetic buffer, respectively. The glycan-loaded biosensors were submerged in wells containing different concentrations of Siglec-10 for 5 min followed by 15 min of dissociation in 1× kinetic buffer at 25°C with the orbital shake speed of 1000 rpm. As a reference control for subtraction, glycan-loaded biosensors were also dipped in wells containing 1× kinetic buffer. The binding kinetics data were processed by the ForteBio data analysis software (version 11.1). The association and dissociation curves were fitted, and the avidity values were calculated by using a heterogeneous ligand (2:1) model.

## Results

### Fine Specificity of Human Siglecs Toward the 98 *N*-Glycan Microarray

The primary glycan ligands of Siglecs were reported and well summarized (Macauley et al., 2014; Duan and Paulson, 2020). Human Siglec-3, -8, -9, and -10 and mouse Siglec-F recognize Neu5Acα2-6Galβ1-4GlcNAc (Ac6LN) and/or Neu5Acα2-3Galβ1-4GlcNAc (Ac3LN), which are often identified as terminal epitopes on complex glycans found on mammalian cells. To explore fine binding specificities of Siglecs, these Siglecs were analyzed against a previously fabricated microarray containing 98 structurally well-defined complex glycans (Supplementary Figure S1) (Li et al., 2019).

**FIGURE 1:**
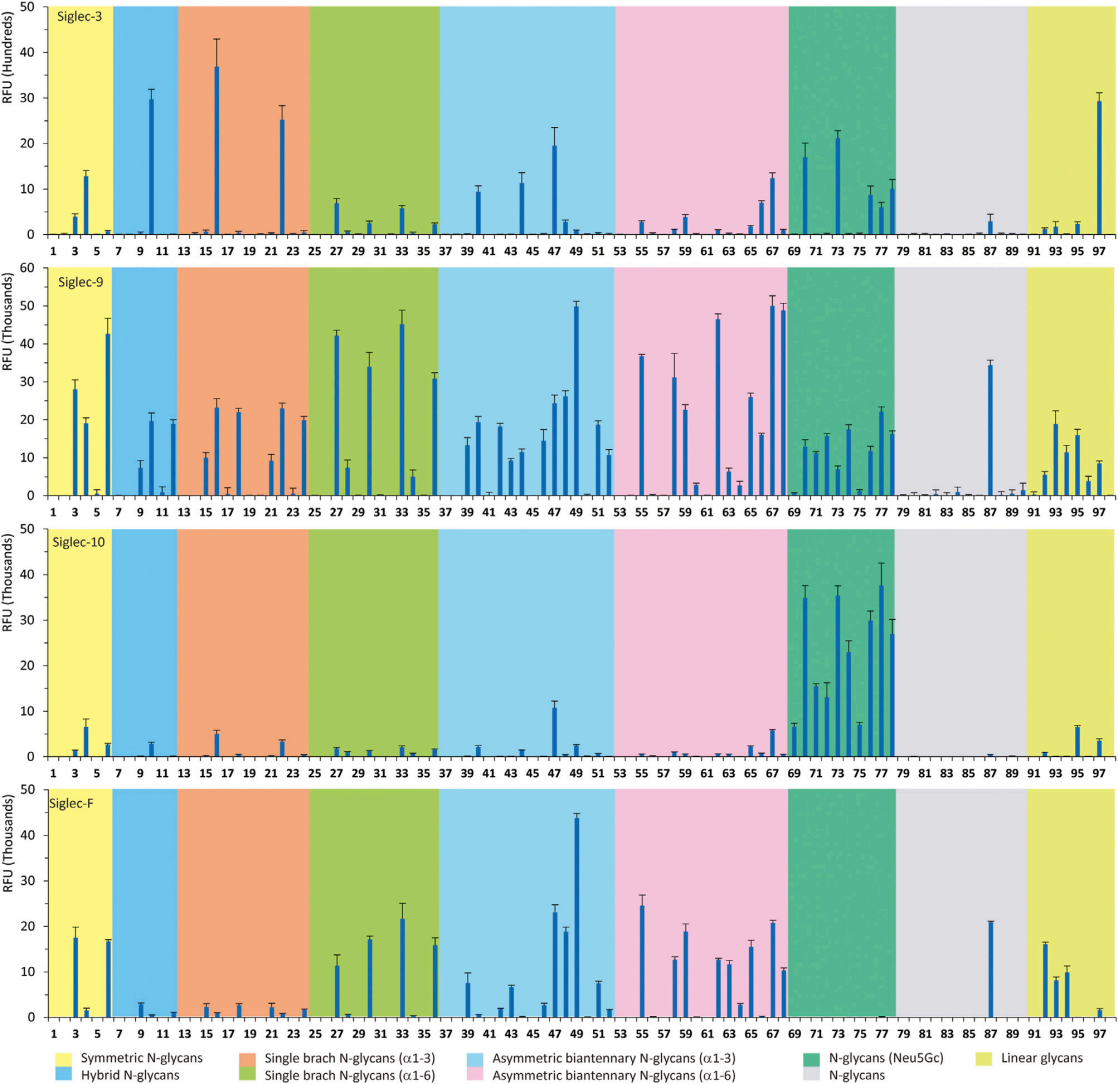
Selective recognition of Sia-containing *N*-glycans by human and mouse Siglecs.

As shown in Figure 1, human Siglec-3 gave lower binding signals toward *N*-glycans compared with Siglec-9, -10, and -F. Siglec-3, which is found on myeloid cells, is associated with acute myeloid leukemia (AML) and Alzheimer’s disease (Freeman et al., 1995; Zhao, 2019) and was reported to prefer the Ac6LN trisaccharide, plus relatively weak affinity to Gc6LN (Neu5Gcα2-3Galβ1-4GlcNAc) and Ac3LN (Blixt et al., 2003). Recently, Rodrigues et al. (2020) reported that Siglec-3 could recognize both α2-3 and α2-6sialosides in solution and on cells. This is consistent with our results that it bound to *N*-glycans with terminal epitopes Ac3LN (compound **3**), Ac6LN (compound **4**), Gc3LN (compound **77**), and Gc6LN (compound **78**). In addition, the RFUs of Siglec-3 to α2-6sialosides (**4** and **78**) are higher than those of α2-3sialosides (**3** and **77**), again consistent with a previous report toward O-mannosyl glycans (Meng et al., 2018). In addition, a slight preference toward Neu5Ac over Neu5Gc was observed, as binding signals of glycans with Neu5Ac residues (**4**, **10**, and **16)** were greater than those of their Neu5Gc-containing counterparts (**70**, **73**, and **78**). Furthermore, high to moderate bindings were observed toward *N*-glycans carrying Ac3LN on the α1-3 branch (**16**, **33**, **40**, **44**, **47**, **66**, and **67**), whereas no meaningful binding signals were observed to their positional isomers (**28**, **34**, **50**, **51**, **56**, **60**, and **63**). These data suggested that Siglec-3 had an apparent preference toward the α1-3 branch when terminal epitope Ac6LN serves as the binding ligand. On the other hand, Siglec-3 exhibited an opposite branch preference toward the α1-6 branch when terminal epitope Ac3LN served as the binding ligand; for example, it bound to **27**, **33**, **55**, and **59**, but failed to bind their positional isomers (**15**, **21**, **39**, and **43**). Such a unique terminal epitope-dependent branch preference was double evidenced by strong binding to **47**, which presents terminal epitopes on preferred branches (Ac6LN on the α1-3 branch and Ac3LN on the α1-6 branch), but no binding to **63** that presents terminal epitopes on nonpreferred branches (Ac3LN on the α1-3 branch and Ac6LN on the α1-6 branch).

Siglec-9 was reported to bind to both α2-3 and α2-6sialosides, with a high affinity to epitope Neu5Acα2-3Galβ1–4(6-sulfo)GlcNAc (Zhang et al., 2000; Rillahan et al., 2012; Duan and Paulson, 2020). Our microarray results are consistent with previous reports as all related *N*-glycans showed binding signals. *N*-glycans with sialyl Lewis X (sLe^X^) epitopes showed the highest binding signals, including compounds **6**, **27**, **33**, **49**, **67**, and **68**. Additionally, glycans with the Ac3LN epitope (**3**, **27**, and **33**) exhibited higher bindings than those with the Ac6LN epitope (**4**, **28**, and **34**). Interestingly, the same terminal epitope-dependent branch preference for Siglec-3 was also observed for Siglec-9. When bound to glycans with the terminal epitope Ac6LN, Siglec-9 showed an apparent preference toward the α1-3 branch (**16**, **22**, **40**, and **44**) over the α1-6 branch (**28**, **34**, **66**, and **60**) of *N*-glycans. In contrast, an opposite branch preference was found when bound to glycans with the terminal epitope Ac3LN and sLe^X^. Lastly, it is observed that Siglec-9 has a slight preference to Neu5Ac-containing *N*-glycans (**9**, **10**, **12**, **15**, **16**, **18**, **39**, and **40**) over their Neu5Gc-containing counterparts (**69–76**).

The specificity of Siglec-10 was previously profiled as having a high affinity to Gc6LN, with moderate and weak affinity to Ac6LN and Ac3LN, respectively (Crocker et al., 2007). In our array, as expected, Siglec-10 exhibited strong bindings to the majority of Neu5Gc-terminated *N*-glycans (**69–78**). In addition, bindings of Siglec-10 toward various glycan ligands showed gradient diminished signals (Gc6LN >> Gc3LN > Ac6LN >> Ac3LN). For example, the binding signals of Siglec-10 to complex type *N*-glycans **76** (Gc6LN) >> **75** (Gc3LC) > **40** (Ac6LC) >> **39** (Ac3LN) and hybrid type *N*-glycans **70** (Gc6LN) >> **69** (Gc3LC) > **10** (Ac6LC) >> **9** (Ac3LN) clearly showed this trend. Siglec-10 also exhibited a terminal epitope-dependent branch preference toward asymmetric *N*-glycans. For example, high to moderate bindings were observed when epitope Ac6LN was presented on the terminal of the α1-3 branch (**16**, **22**, **66**, **40**, and **44**), while no meaningful signals could be observed to their positional isomers where Ac6LN was presented on the α1-6 branch (**28**, **34**, **50**, **56**, and **60**). This result suggested that Siglec-10 had an apparent preference toward the α1-3 branch when α2-6sialylated glycans served as ligands. In contrast, Siglec-10 exhibited an opposite branch preference toward the α1-6 branch when terminal epitope Ac3LN or sLe^X^ served as the binding ligand. This is evidenced by relatively weak bindings toward compounds **27**, **30**, **33**, and **36**, which present Ac3LN or sLe^X^ on the α1-6 branch but no bindings to their positional isomers. This terminal epitope-dependent branch preference is identical to that of Siglec-3 and Siglec-9.

Human Siglec-8 did not bind to any glycans on the array (data not shown), which is consistent with previous observations that Siglec-8 specifically recognizes Neu5Acα2-3 (6-sulfo)Galβ1–4GlcNAc (6-sulfo-sLe^X^) (Bochner et al., 2005). Mouse Siglec-F is a functional paralogue of human Siglec-8, and it was reported to bind to Ac3LN and 6'-sulfo-sLe^X^ (Tateno et al., 2005). As depicted in Figure 1, Siglec-F could recognize *N*-glycans with Ac3LN and sLe^X^ epitopes, such as **3**, **6**, and **49**, whereas no binding was observed toward any Neu5Gc-containing glycans, suggesting a strict preference toward Neu5Ac. In addition, Siglec-F showed an apparent α1-6 branch preference. For example, high binding signals were observed for glycans **27**, **30**, **33**, and **36**, but very low bindings were observed for their positional isomers **15**, **18**, **21**, and **24**. Interestingly, compound **49**, which contains sLe^X^ on the α1-3 branch and Ac3LN on the α1-3 branch, showed the strongest binding signals.

### Chemoenzymatic Synthesis of Neu5Gc-Containing *N*-Glycans

**FIGURE 2:**
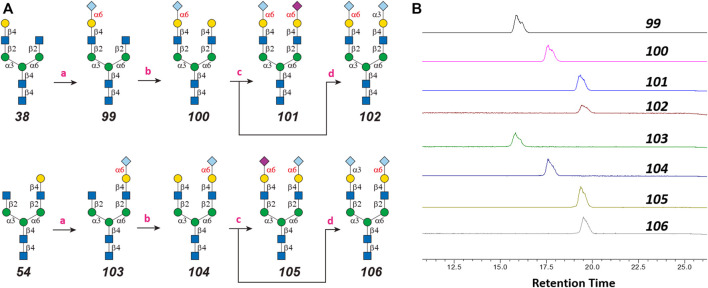
Enzymatic synthesis of Neu5Gc-containing asymmetric *N*-glycans: **(A)** a, α2-6sialylation with Pd26ST, NmCSS, CTP, and Neu5Gc; b, β1-4galactosylation with NmLgtB and UDP-Gal; c, α2-6sialylation with Pd26ST, NmCSS, CTP, and Neu5Ac; d, α2-3sialylation with PmST1-M144D, NmCSS, CTP, and Neu5Gc; **(B)** HPLC analysis of purified *N*-glycans.

One interesting observation is that Siglec-10 showed high binding to an asymmetric *N*-glycan **47** but no binding to its positional isomer **63** (Figure 1). Such a dramatic binding divergence can be explained by its terminal epitope-dependent branch preference, as both ligands (Ac3LN and Ac6LN) on **47** are located on the terminal of favored branches, whereas both ligands are located on the unfavored branches of **63**. Because Siglec-10 strongly prefers Neu5Gc-containing *N*-glycans, we speculate that a Neu5Gc-modified counterpart of 47 (Figure 2A, compound **102**) may be of higher affinity and a most favorable *N*-glycan ligand of Siglec-10. To test this hypothesis and to further validate the terminal epitope-dependent branch preference of Siglec-10, we enzymatically synthesized eight Neu5Gc-containing *N*-glycans (Figure 2A). In detail, compounds **99** to **102** were assembled starting from previously prepared glycan **38** (Li et al., 2015). First, α2-6Neu5Gc was installed onto the α1-3 branch to achieve **99** by Pd26ST-catalyzed α2-6sialylation in the presence of cytidine-5′-triphosphate (CTP), Neu5Gc, and NmCSS for the *in situ* generation of the sugar donor CMP-Neu5Gc. Then, β1-4Gal was installed onto the α1-6 branch by NmLgtB-catalyzed reaction in the presence of UDP-Gal to provide **100**. The addition of α2-6Neu5Ac to the α1-6 branch of **100** by Pd26ST then provided **101**. On the other hand, the addition of α2-3Neu5Gc to this branch by PmST1-M144D-catalyzed α2-3sialylation gave the desired asymmetric *N*-glycan **102**. In the same synthetic manner, another four asymmetric *N*-glycans **103**, **104**, **105**, and **106** were assembled starting from *N*-glycan **54**. All compounds were purified and characterized by HPLC (Figure 2B), mass spectrometry, and NMR (supporting information).

### Neu5Gc *N*-Glycan Microarray Fabrication and Assay With Human Siglec-10

**FIGURE 3:**
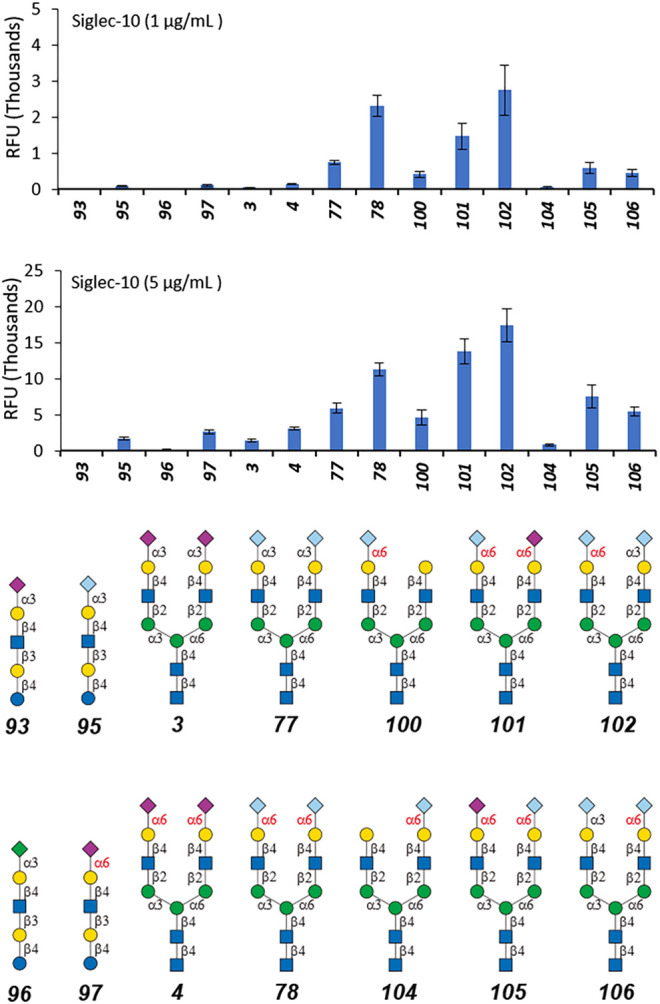
Selective recognition of Neu5Ac-containing *N*-glycans by human Siglec-10.

The Neu5Gc-containing *N*-glycans were labeled with AEAB to provide an amino group for microarray fabrication as previously reported (Li et al., 2019). The Neu5Gc *N*-glycan microarray was then constructed on NHS glass slides with four additional sialylated *N*-glycans (**3**, **4**, **77**, and **78**) and four sialylated linear glycans (**93**, **95**, **96**, and **97**). The recognition by Siglec-10 was then assayed at the concentrations of 1 µg/ml and 5 µg/ml (Figure 3). As shown in Figure 3, di-sialylated glycans (**78**, **101**, **102**, and **105**) showed high RFU compared with mono-sialylated glycans (**100**and **104**) and linear glycan (**93**, **95**, **96**, and **97**). The preferences of Siglec-10 toward Neu5Gc (**77**, **78**) over Neu5Ac (**3** and **4**) and α2-6sialosides (**4**, **78**) over α2-3sialosides (**3**, **77**) were further confirmed by this focused array. In addition, when terminal epitope Gc6LN served as the binding ligand, Siglec-10 preferred the α1-3 branch (**100**) over the α1-6 branch (**104**). And as expected, Siglec-10 showed the highest binding signal to glycan **102** (the Neu5Gc modified counterpart of **47**), whereas its positional isomer **106** only showed comparable bindings as that of mono-sialylated **100**. These results further confirmed the terminal epitope-dependent branch preference; that is, Siglec-10 prefers α2-6sialosides on the α1-3 branch and α2-3sialosides on the α1-6 branch of *N*-glycans. Interesting, the binding signals of Siglec-10 toward the four α2-6sialyated *N*-glycans with Neu5Ac/Neu5Gc chimeras (**4**, Neu5Ac on both branches; **78**, Neu5Gc on both branches; **101**, Neu5Ac on α1-6 branch, Neu5Gc on α1-3 branch; **105**, Neu5Gc on α1-6 branch, and Neu5Ac on α1-3 branch) are distinct, indicating that minor structural divergence in complex glycan may cause substantial changes in glycan-protein interactions.

### Avidity of Siglec-10 to *N*-Glycans **102** and **106**

**FIGURE 4:**
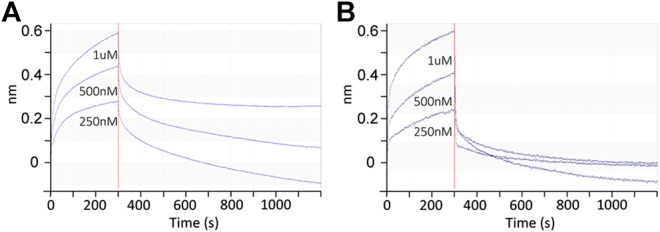
Binding kinetics between the Siglec-10-Fc chimera protein homodimer and Neu5Gc-containing *N*-glycans **102 (A)** and **106 (B)** determined by BLI. Association and dissociation phases are shown and separated by the red dashed line at 300 s.

As shown in Figure 3, the binding signals of Siglec-10 to **102** is around 5-fold stronger than to its positional isomer **106** and 20-fold stronger than to linear glycans **97**, suggesting compound **102** as a potential high-affinity ligand of Siglec-10. The avidity of Siglec-10 toward **102** and **106** was thus measured by biolayer interferometry (BLI). AEAB labeled **102** and **106** were further conjugated with NHS-Biotin and purified with HPLC, and then immobilized onto streptavidin-coated biosensors for BLI assay (Figure 4). The association and dissociation curves were fitted, and the avidity values were calculated with the consideration of the bivalency of the Siglec-10-Fc chimera protein. The avidity values of Siglec-10 toward **102** and **106** were 0.11 μM and 7.34 μM, respectively, indicating a 67-fold higher avidity of **102** than **106**. The result further confirmed the terminal epitope-dependent branch preference and revealed a high avidity glycan-binding partner (**102**) of human Siglec-10.

## Discussion and Conclusion

Siglecs are attractive therapeutic targets and several related antibody-based therapies had been developed for the treatment of immune-related diseases. In certain applications, glycan ligands have an advantage over antibodies, such as their ability to dissociate from their target once endocytosed. However, glycan-based therapeutic strategies for cargo delivery and immunomodulation are underinvestigated due to the lack of suitable ligands (Angata et al., 2015). A comprehensive understanding of glycan recognition details by Siglecs is essential toward the discovery and designing of efficient ligands. In fact, recent advances in glycobiology have prompted such applications. For example, high specific efficient *N*-glycan ligands with chemical modifications toward Siglec-2 were reported (Peng and Paulson, 2017). Conjugates of toxins with this novel ligand could be efficiently internalized *via* Siglec-2, resulting in the killing of B-cell lymphoma cells.

In this study, we screened binding profiles of Siglec-3, -9, -10, and -F against a comprehensive *N*-glycan microarray to reveal glycan recognition details of Siglecs (Table 1). The results showed a surprising terminal epitope-dependent branch preference toward *N*-glycans by Siglec-3, -9, and -10. These Siglecs prefer the α1-3 branch of *N*-glycans when α2-6sialylated epitopes serve as binding ligands, while they have an opposite preference to the α1-6 branch when α2-3sialylated epitopes serve as ligands. Such a feature could assist in designing high-affinity binding partners of Siglecs. For example, we designed and synthesized an asymmetric *N*-glycan (102) with much higher avidity than its positional isomer toward Siglec-10. Note that recombinant Siglec-Fc chimera proteins in the form of disulfide-linked homodimer were used in this study instead of native Siglecs. Even though such chimera proteins were widely used to reveal the glycan recognition of Siglecs and other human GBPs (Blixt et al., 2003; Bochner et al., 2005; Campanero-Rhodes et al., 2006; Rillahan et al., 2012; Rillahan et al., 2013; Gao et al., 2019b; Rodrigues et al., 2020), the nonnatural bivalent form could possibly influence their fine specificity toward glycan-binding partners.

**TABLE 1:** Binding specificity of Siglecs toward *N*-glycans observed in this study. Recombinant Siglec-Fc chimera proteins were used in this study.

Siglec	Ligand (preference)	Branch Preference	Strongest *N*-glycan Binder
Siglec-3	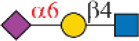 _(+++);_ 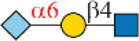 _(+++)_	α1-3 branch	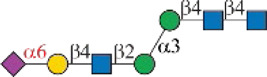
	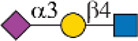 _(++)_	α1-6 branch	
Siglec-9	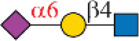 _(+++);_ 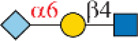 _(++)_	α1-3 branch	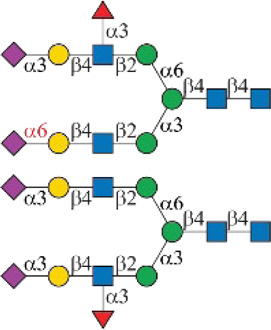
	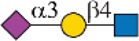 _(+++);_ 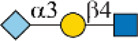 _(++)_	α1-6 branch	
	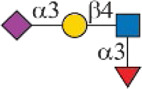 _(+++);_ 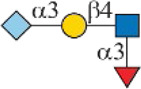 _(++)_	α1-6 branch	
Siglec-10	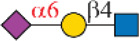 _(+);_ 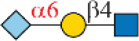 _(+++)_	α1-3 branch	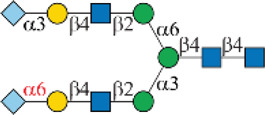
	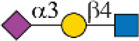 _(weak);_ 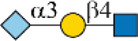 _(++)_	α1-6 branch	
	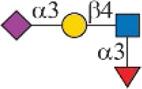 _(weak);_ 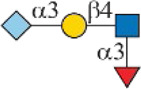 _(++)_	α1-6 branch	
Siglec-F	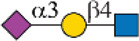 _(++);_ 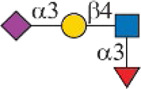 _(++)_	α1-3 branch	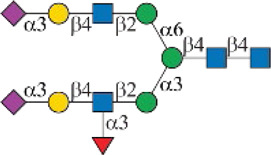
Symbols: 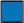 GlcNAc 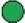 Man 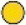 Gal 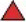 L-Fuc 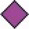 Neu5Ac 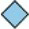 Neu5Gc			

High-avidity binding partners of Siglecs could lead to extensive academic and clinical implementations. For example, tumor cells can escape the surveillance of the immune system *via* inhibition of immune cells through immune checkpoints and their ligands. A promising therapeutic approach for cancer is to block these immune checkpoints, for example, the programmed cell death ligand 1 (PD-L1) and cytotoxic T lymphocyte-associated protein 4 (CTLA-4) (Leach et al., 1996; Topalian et al., 2012). A recent report showed that CD24–Siglec-10 interaction is an innate immune checkpoint that is essential for mediating antitumor immunity and can promote tumor immune escape. The modulation of this interaction is expected to become a new target for tumor therapy (Barkal et al., 2019). The high sialylated CD24 that overexpressed on tumor cells functions as the main ligand of Siglec-10. It induces the inhibition of the immune system and promotes tumor immune escape. Additionally, CD24–Siglec-10 interaction could suppress the immune response to the danger-associated molecular pattern (DAMP) (Cai et al., 2009; Rillahan et al., 2012). It is thus tempting to speculate that the strong Siglec-10 binding partner **102**, with or without further modification, may serve as an invaluable reagent to block this immune checkpoint.

## Data Availability

The original contributions presented in the study are included in the article/Supplementary Material, further inquiries can be directed to the corresponding author.
