# Supplementary material for: Terminal Epitope-Dependent Branch Preference of Siglecs Toward N-Glycans
Source: Front Mol Biosci. 2021 Apr 29;8:645999. doi: 10.3389/fmolb.2021.645999 (PMC8116747; doi:10.3389/fmolb.2021.645999)
Supplement: Supplementary file 1 [file DataSheet1.PDF]

Siglec-10 bind asymmetric sialic acid-containing N-glycans with high avidity

Shuaishuai Wang,[1∇] Congcong Chen,[1∇] Minhui Guan,[2,3,4] Ding Liu,[1] Xiu-Feng Wan,[2,3,4,5] Lei Li[1*]

[1] Department of Chemistry, Georgia State University, Atlanta, GA, USA

[2]MU Center for Research on Influenza Systems Biology (CRISB), University of Missouri, Columbia, Missouri, 65211, USA

[3]Department of Molecular Microbiology and Immunology, School of Medicine, University of Missouri, Columbia, Missouri, 65211, USA

[4]Bond Life Sciences Center, University of Missouri, Columbia, Missouri, 65211, USA

[5]Department of Electrical Engineering & Computer Science, College of Engineering, University of Missouri, Columbia, Missouri, 65211, USA

Correspondence:
Lei Li
lli22@gsu.edu

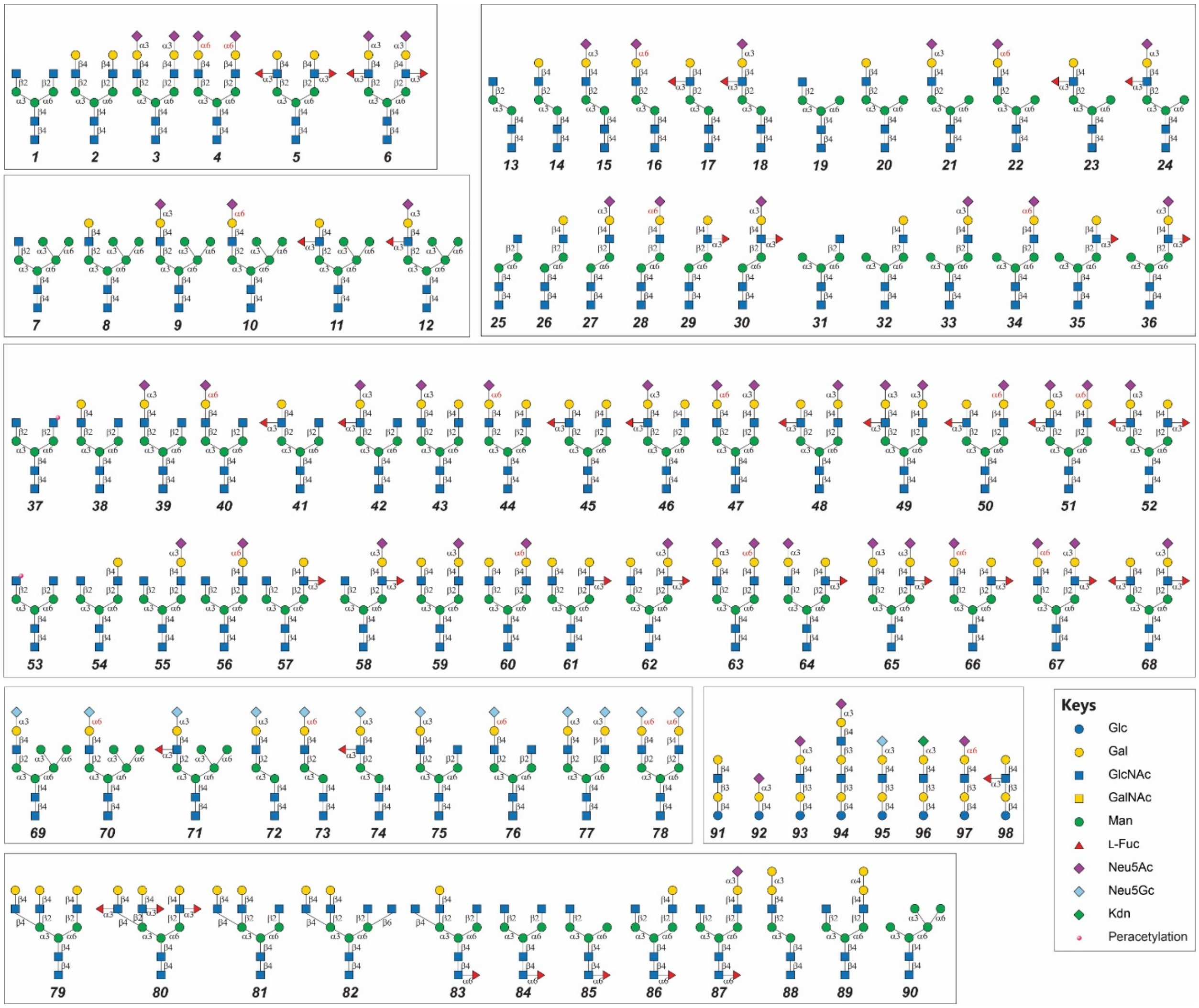

Figure S1. Structures of all glycans printed in the glycan microarray. The symbolic nomenclature for glycans is shown.

III. NMR analyses of *N*-glycans prepared in this study

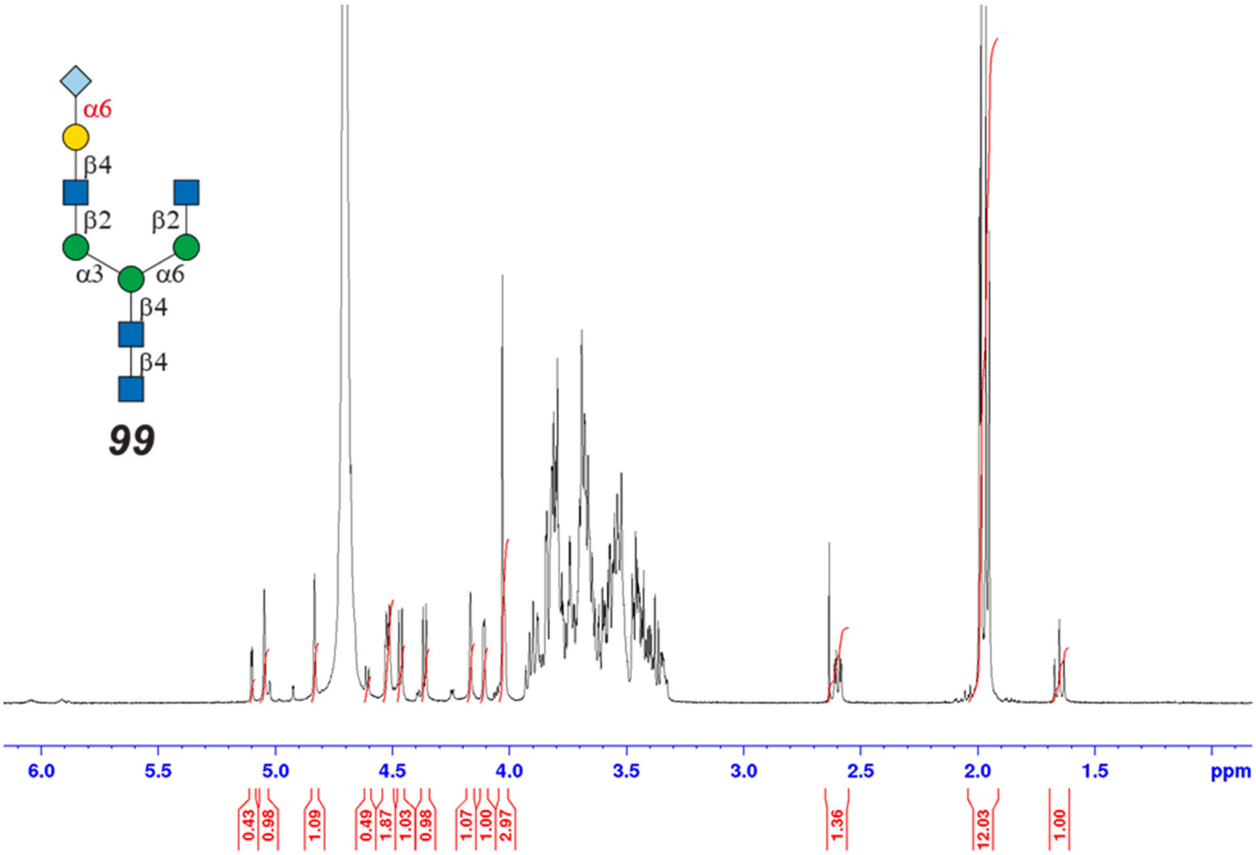

^{1}H NMR of compound **99**

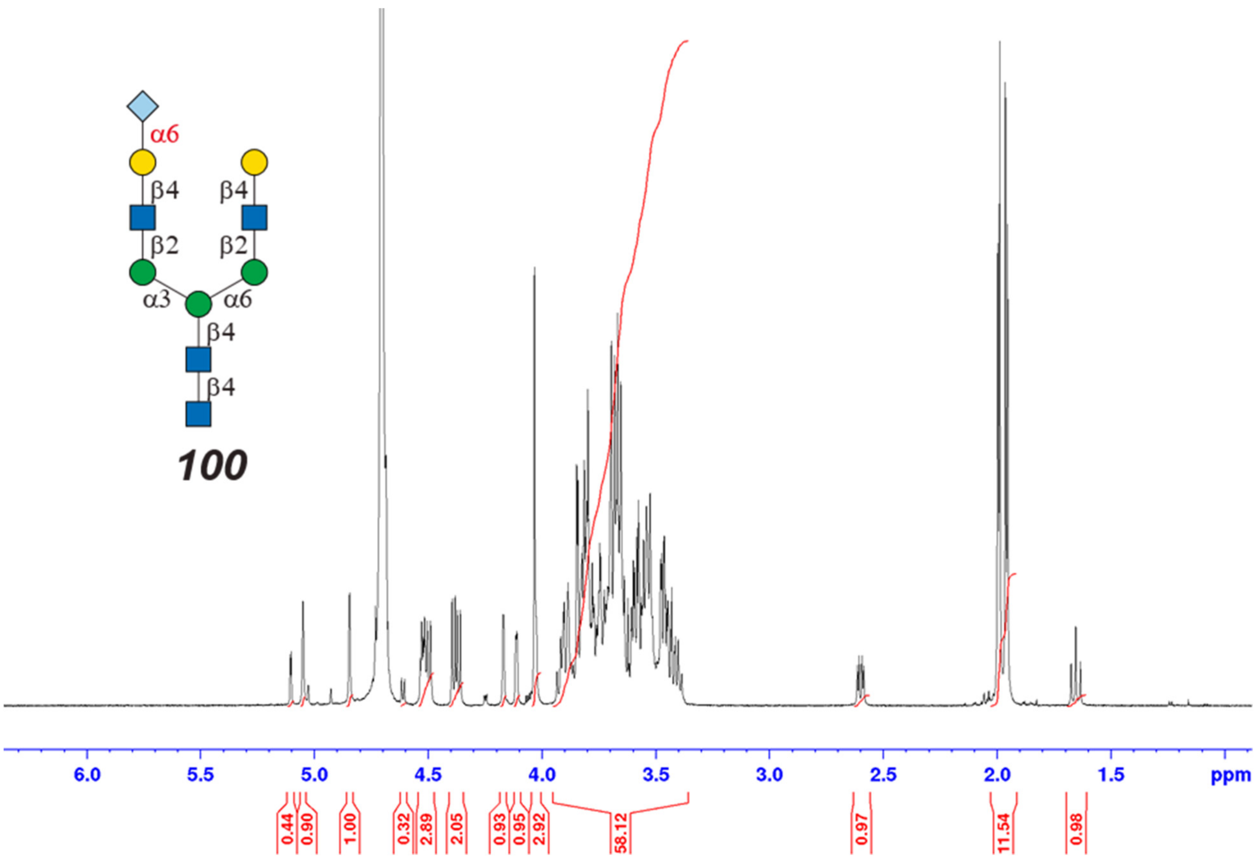

^{1}H NMR of compound **100**

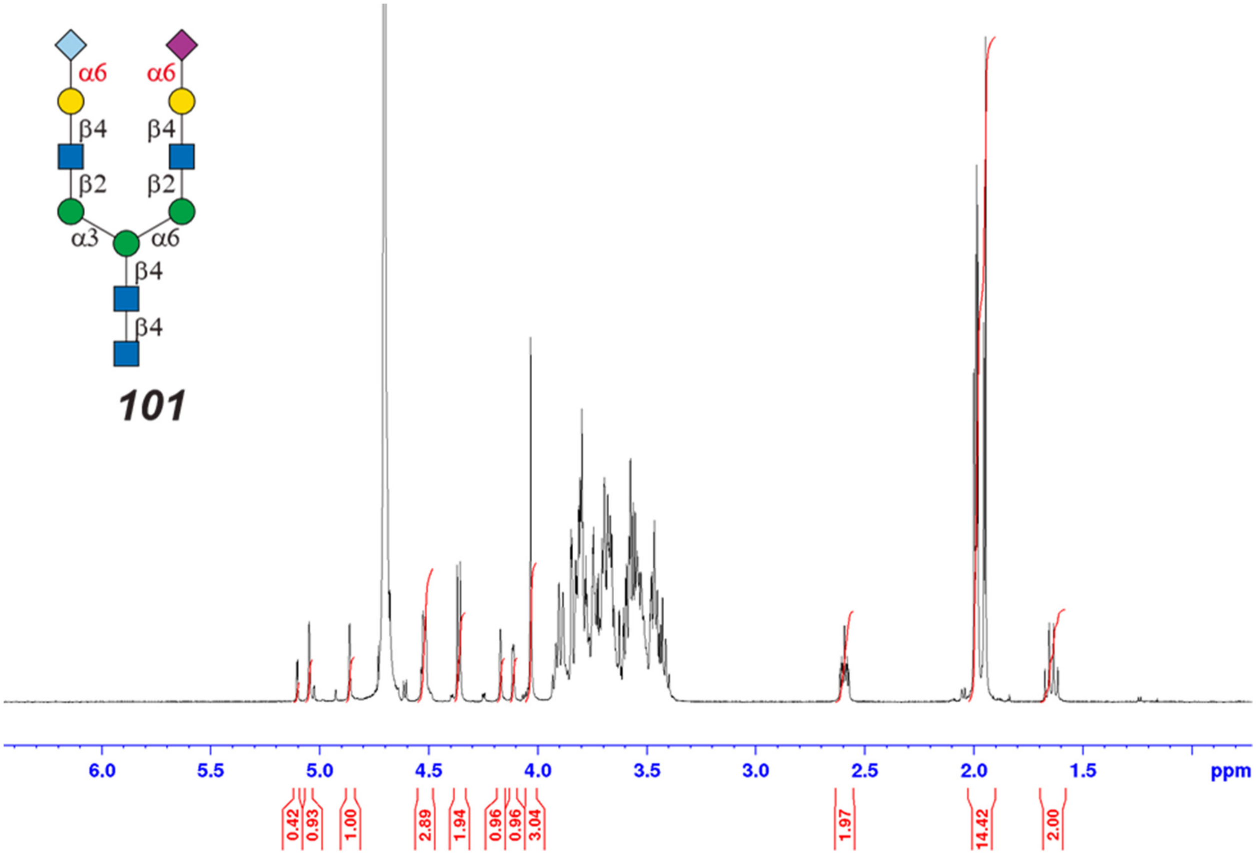

^{1}H NMR of compound **101**

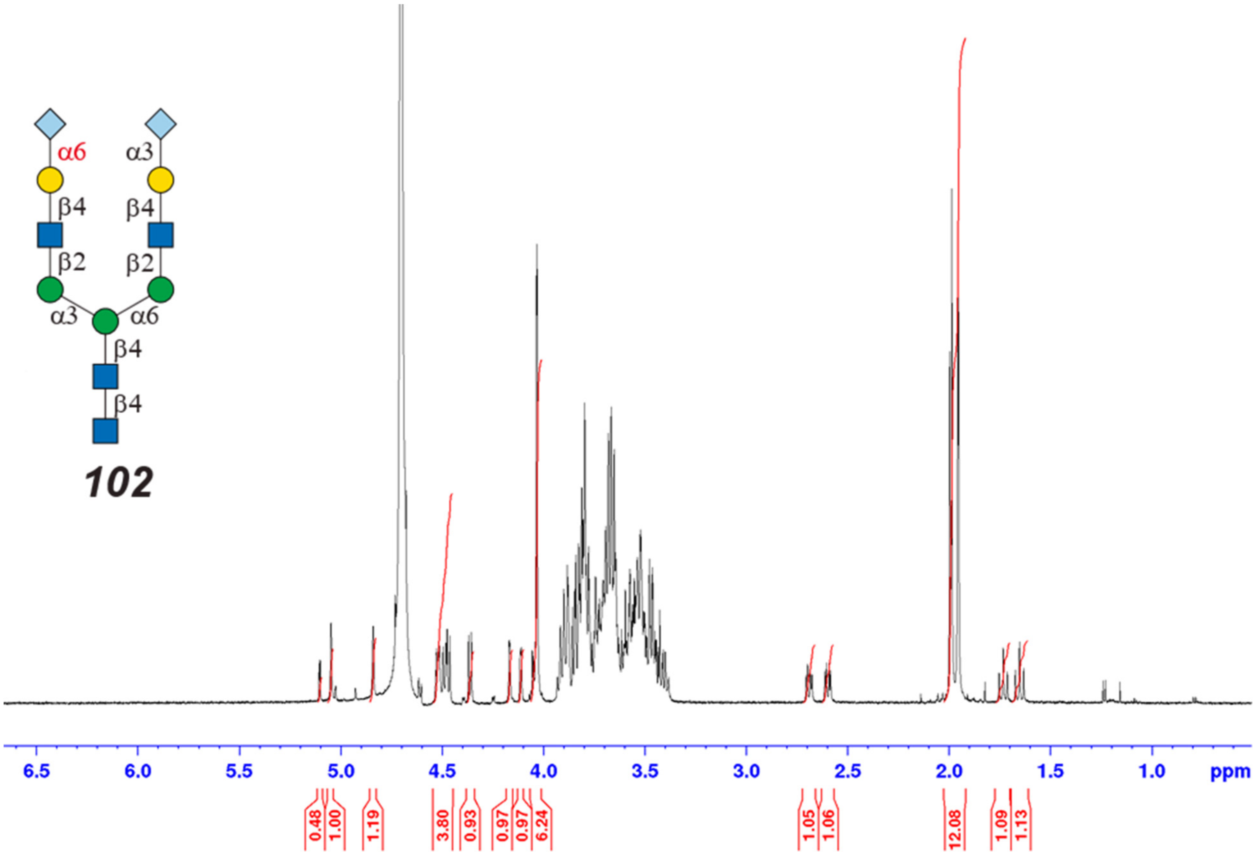

^{1}H NMR of compound **102**

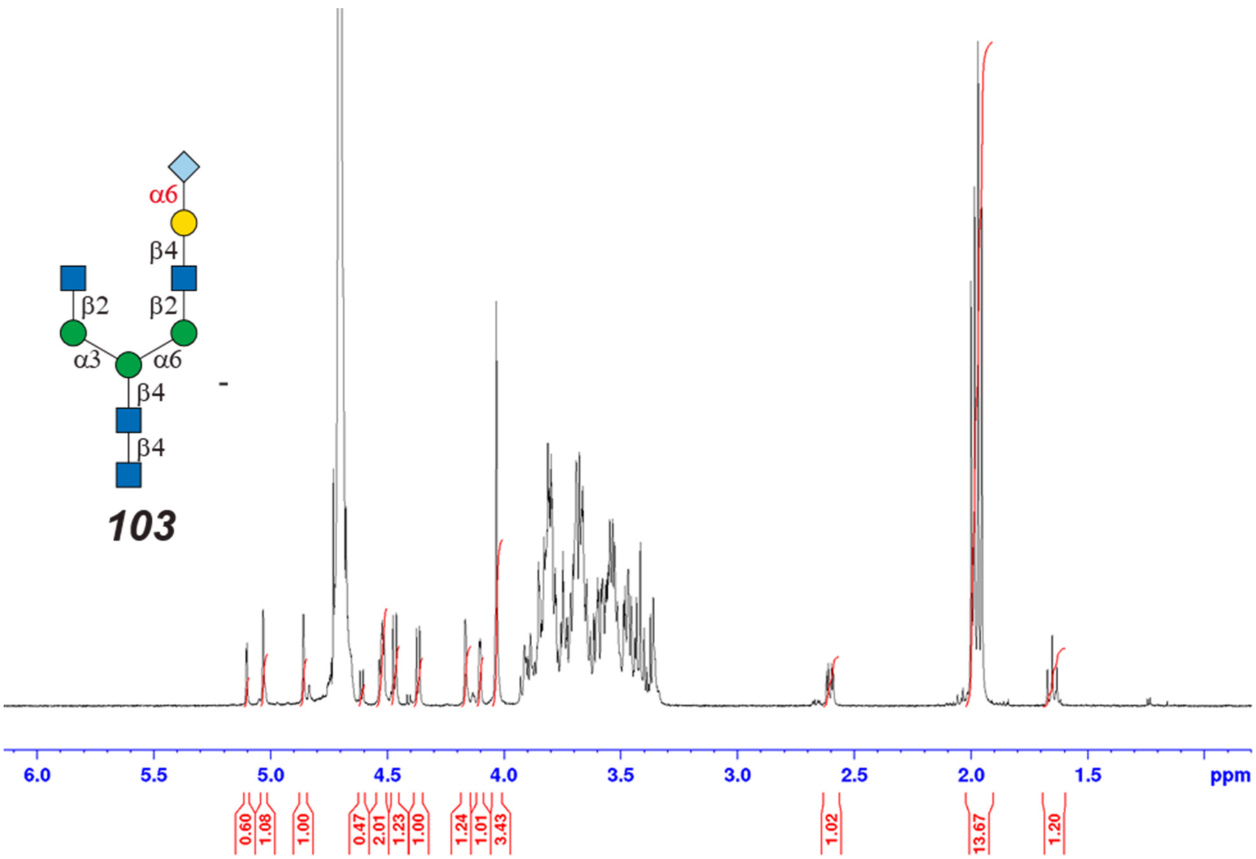

^{1}H NMR of compound **103**

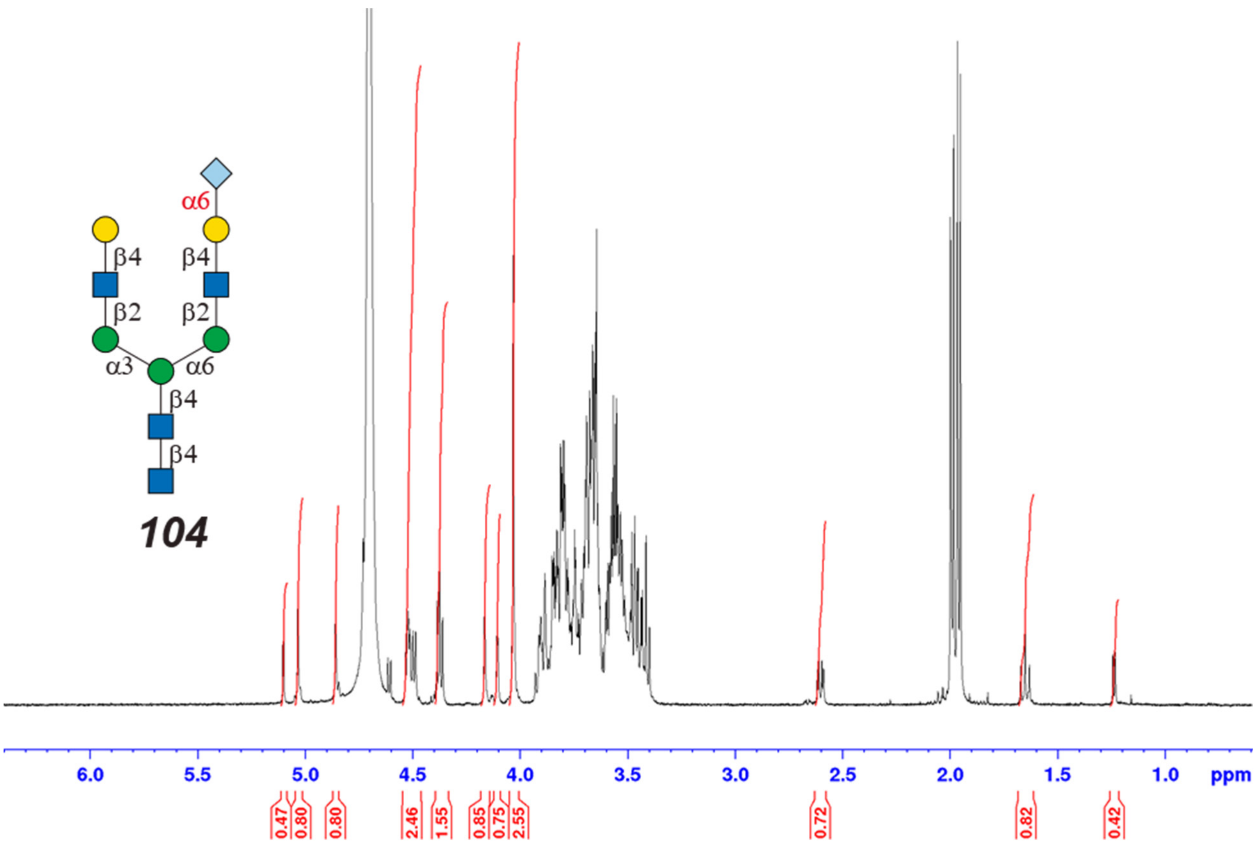

104

^{1}H NMR of compound **104**

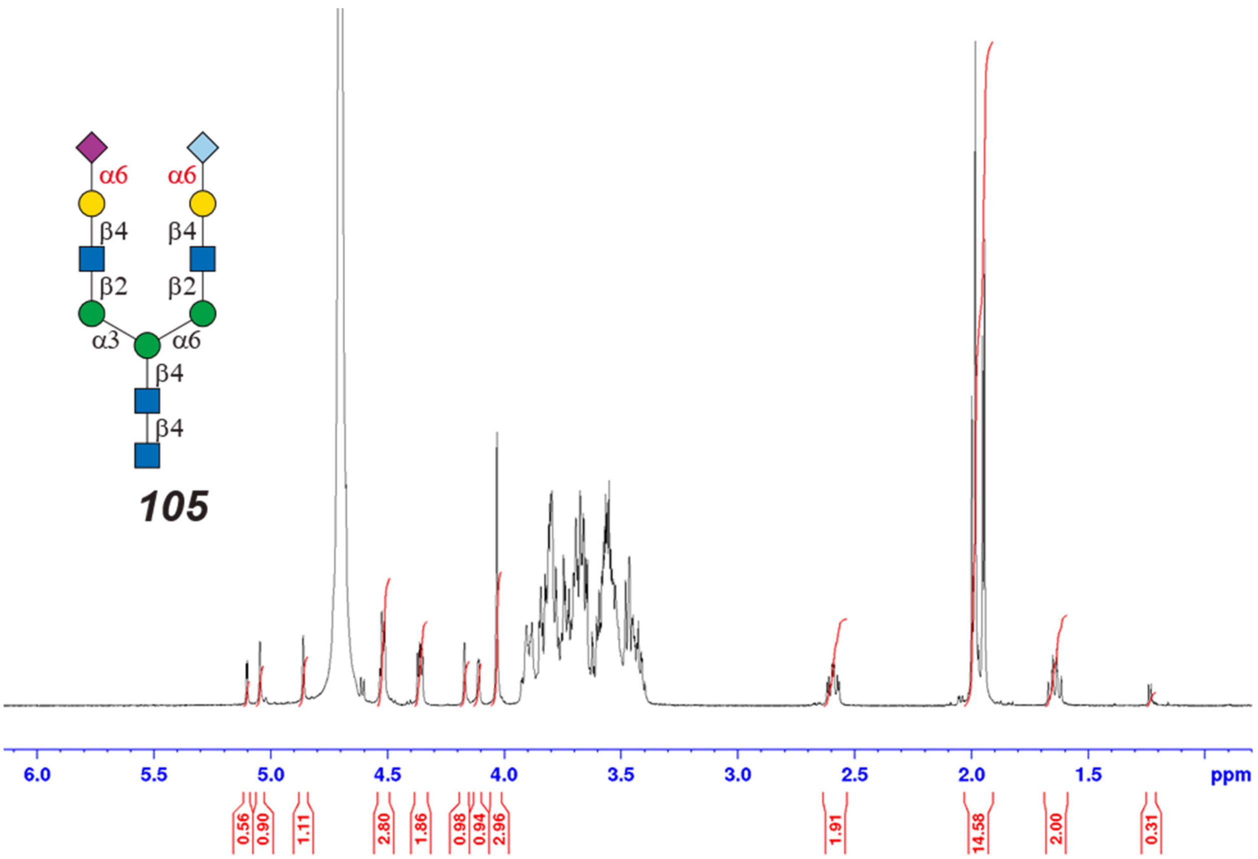

^{1}H NMR of compound **105**

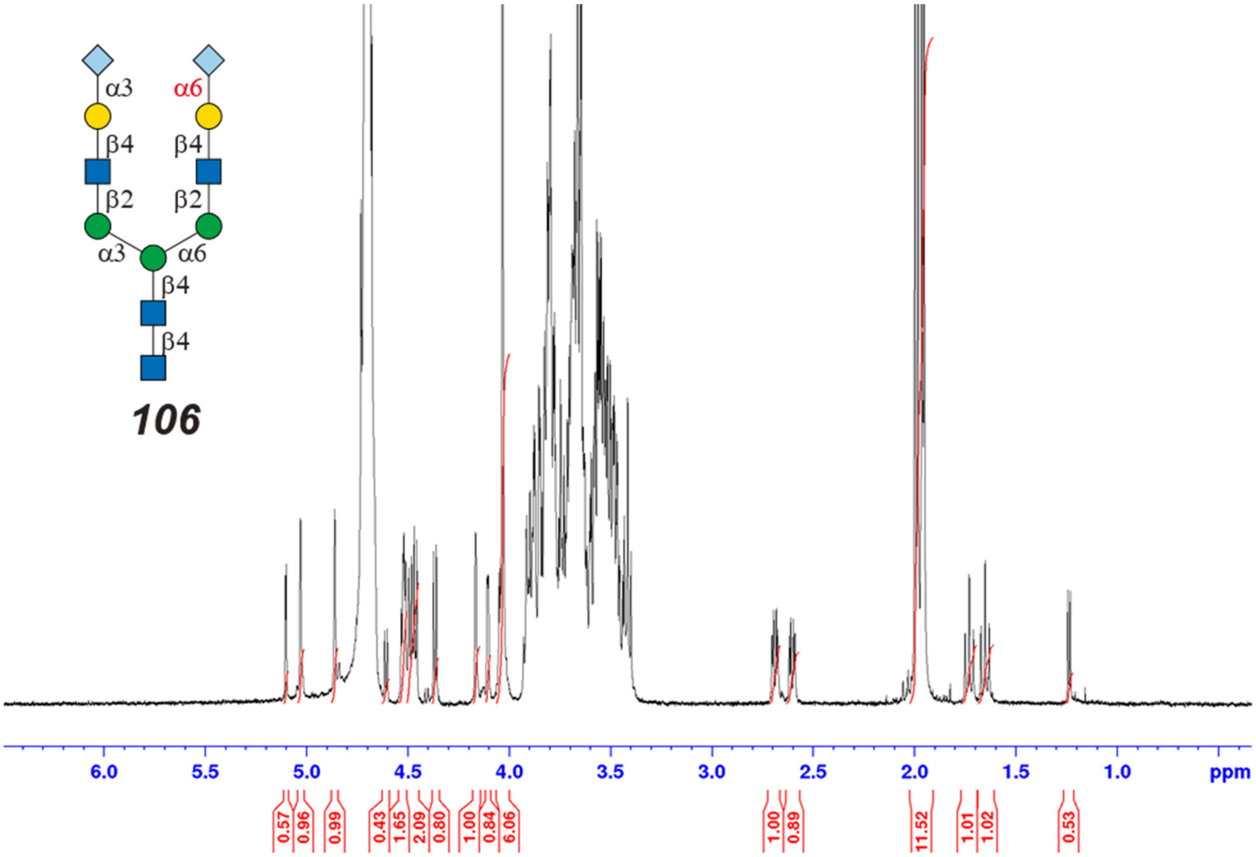

^{1}H NMR of compound **106**
